# Are there differences in revision stapes surgery outcomes between university and county clinics? A study from the quality register for otosclerosis surgery in Sweden

**DOI:** 10.1007/s00405-022-07737-5

**Published:** 2022-11-11

**Authors:** Nina Pauli, Caterina Finizia, Lars Lundman, Andreas Björsne, Ylva Dahlin-Redfors

**Affiliations:** 1 Department of Otorhinolaryngology, Institute of Clinical Sciences, Sahlgrenska University Hospital/Sahlgrenska, Sahlgrenska Academy at the University of Gothenburg, Gothenburg, Sweden; 2 Department of Otorhinolaryngology, Region Västra Götaland, Sahlgrenska University Hospital, Gothenburg, Sweden; 3 Department of Otolaryngology, Central Hospital, Karlstad, Sweden; 4 Region Västra Götaland, Habilitation and Health, Hearing Organization, Gothenburg, Sweden; 5 Unit of Audiology, Institute of Neuroscience and Physiology, Sahlgrenska Academy at the University of Gothenburg, Gothenburg, Sweden

**Keywords:** Otosclerosis, Stapedotomy, Revision surgery, Quality register, Outcome

## Abstract

**Purpose:**

The aim of the study was to investigate hearing outcomes in stapes revision surgery with regard to the type of clinic (university clinic or county clinic). Furthermore, the aim was to investigate the risk of complications with a focus on tinnitus, hearing deterioration, and taste disturbance 1 year after surgery.

**Methods:**

The study is based on data from the Swedish Quality Register for Otosclerosis Surgery (SQOS). Two study protocols were completed by the surgeon, and a questionnaire was distributed to the patients 1 year after surgery. A total of 156 revisions were available for analysis with both preoperative and postoperative audiometry data.

**Results:**

Seventy-five percent of the patients reported better to much better hearing 1 year after revision surgery. An air bone gap ≤ 20 dB postoperatively was seen in 77% of the patients. Four percent had hearing deterioration ≥ 20 dB PTA_4_ AC. Eleven percent had worsened or newly developed tinnitus, 5% had taste disturbance, and 3% had dizziness 1 year after surgery. Preoperative and postoperative hearing did not differ between patients operated on in university vs. county clinics.

**Conclusions:**

Revision surgery in otosclerosis is a challenge for otologists, but no differences in hearing outcomes between university and county clinics were found in this nationwide study. The risk of hearing deterioration and deafness is higher than in primary stapes surgery, and revision surgery should be recommended primarily in cases with a large air–bone gap and moderate to severe preoperative hearing loss.

## Background

Revision surgery for otosclerosis is known to be less successful than primary surgery in terms of hearing outcome [1–3]. In addition, the risk of deteriorating sensorineural hearing is higher with revision surgery than with primary stapes surgery [4–6]. One of the reasons for this is that the technical aspects of the surgical procedure are much less predictable than those of primary stapes surgery. Revision surgery for otosclerosis is often claimed to be one of the most technically challenging procedures in middle ear surgery [2, 6]

In other fields of medicine, such as cancer surgery and cardiovascular procedures, certain surgical procedures of either a higher level of technical difficulty or procedures seldom performed are advised to be performed at highly specialized centers to lower the risks and ensure the best results possible [7–9]. In otorhinolaryngology, earlier studies have shown that high-volume centers have better outcomes in terms of survival for head and neck surgery on the whole and for head and neck cancer surgery more specifically [10–12]. For otological procedures, however, not much research has been performed. A few studies have been conducted on vestibular schwannoma surgery, but the study results are conflicting regarding center volume and its impact on treatment outcome and complications [13, 14]^.^ For middle ear surgery, few studies specifically aiming at investigating these aspects exist.

In Sweden, stapes revision surgery is performed in 18 different clinics. The surgical volumes differs a lot between larger university clinics and smaller county clinics. A study by Yung et al. investigated learning curve in stapes surgery and revealed a relation between high volume of surgical procedures per surgeon and better hearing outcome [15]. By contrast, Strömbäck et al. could find no such differences in stapes surgery outcome between high and low volume surgeons when analysing the national-based register in Sweden [16].

In line with the discussions on centralization and development of highly specialized centers for certain surgical procedures, we aimed to investigate whether the results from the technically challenging surgical procedure of revision stapes surgery in otosclerosis reveal any differences between university clinics and county clinics.

Specifically, the aims of the study were to investigate hearing outcomes in revision surgery for otosclerosis using a nationwide quality register database and to investigate hearing outcomes with regard to the type of clinic (university clinic or county clinic). Furthermore, the study was also intended to investigate the risk of complications after revision surgery with a focus on tinnitus, hearing deterioration, and taste disturbance 1 year after completed surgery.

## Materials and methods

The study is based on prospectively collected data from the Swedish Quality Register for Otosclerosis Surgery (SQOS). SQOS is a nationwide quality register with an estimated coverage of 89% of stapes surgeries performed in Sweden [16, 17]. Patients operated on with revision surgery from 2013 to 2019 were included in the study. Revision surgery was performed at 18 clinics, 10 county clinics and 8 university clinics. Patients with previous revision surgery in the same ear were not included in the study, and only first revisions were included for analysis. Two patients had revisions on both the left and right ears.

The register data are based on two study protocols completed by the surgeon and one patient questionnaire distributed 1 year after surgery. The surgeon completed one protocol on the day of surgery and the other at the 1-year follow-up visit. The protocols include preoperative and postoperative data, including age, sex, type of surgical procedure and method, type of anesthesia, pure tone audiometry, any complications, and data on an earlier surgery or contralateral procedure. The questionnaire to the patient included questions regarding tinnitus, patient satisfaction, hearing level, use of hearing aids and experienced postoperative complications.

### Audiometry

Pure tone audiometry with threshold determination was collected at frequencies of 0.25, 0.5, 1, 2, 3, 4, 6, and 8 kHz. Nonmeasurable thresholds were registered as 130 dB HL (AC) and 75 dB HL (BC). The results regarding the operated ear were presented with pure tone average (PTA_4_) calculated using 0.5, 1, 2, and 4 kHz for air conduction (AC) and bone conduction (BC). The air bone gap (ABG) was calculated by subtracting the PTA4 BC from the PTA4 AC for each subject in the same audiogram according to guidelines for reporting of air–bone gap closure [18, 19]. Grading of hearing loss on the operated ear according to the World Health Organization (WHO) was applied [20]. Notably, in the study the grading refers to the hearing loss of the operated ear and not the best hearing ear.

### Statistical methods

For descriptive variables, the frequency is expressed as the mean and standard deviation for normally distributed data and as the median and range for non-normally distributed data unless otherwise stated. Audiometry data are presented as the median and interquartile range (IQR). Logistic regression analyses were performed with the type of clinic (county clinic or university clinic) as a predictor for postoperative complications and postoperative hearing outcome, and a regression analysis was performed using Fisher’s permutation test. All tests were two-tailed and conducted at the 5% significance level.

### Ethics

The study was approved by the Ethical Review Board in Sweden and performed in accordance with the Declaration of Helsinki.

## Results

**Fig. 1:**
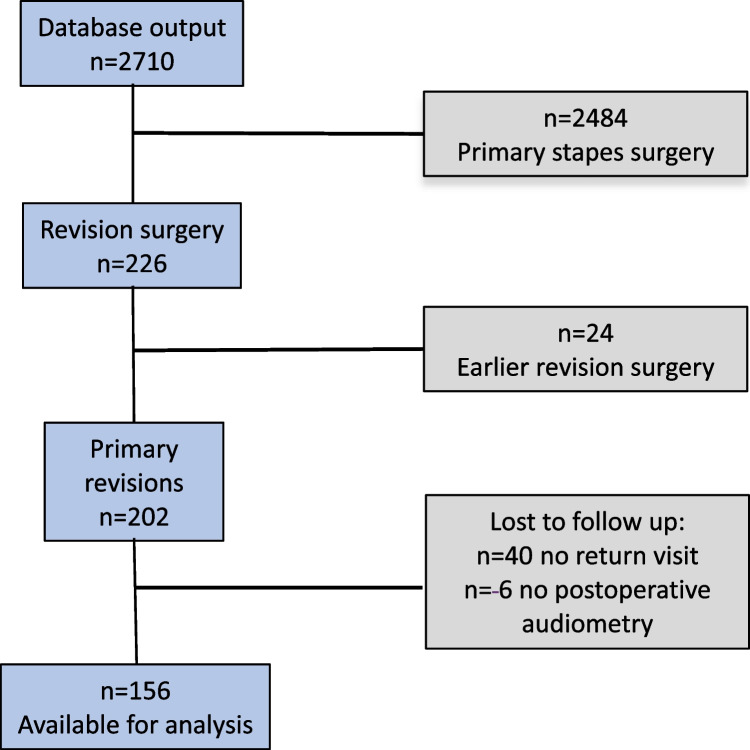
Study flow chart

For this particular study, patients operated on during 2013–2019 were included for analysis. Out of 2710 registered otosclerosis operations, revision surgery constituted 226 surgical procedures, 202 were primary revisions and 156 of them were available for analysis with both pre- and postoperative audiometry. The study questionnaire was completed by 109 patients (Fig. 1). Ninety-five patients had both postoperative audiometry and a completed patient questionnaire.

### Study group and surgical procedure data

The mean age of the study patients was 55.2 years, and 65% were women. Eighty-nine percent were reoperated on with stapedotomy, and 8% were reoperated on with stapedectomy. The majority of the patients were operated on at a university clinic (*n* = 112, 72%). The mean (min–max) number of revision operations per hospital was 14 (1–39) for university clinics and 4.4 (1–13) per clinic for county clinics. Various surgical techniques were applied at the different clinics. A micro burr was used during the procedure in 72 cases (46%), and a laser was used in 55 cases (35%), whereas neither a micro burr nor a laser was applied in 49 cases (31%). Teflon/Platina prostheses were the most common prostheses used (77%), the prosthesis diameter was 0.4 mm in 92% of the cases and 68.6% of the patients were operated with a prosthesis length of 5 mm or shorter.

A majority (76%) were operated on under general anesthesia. Out of 117 cases where the surgeon reported whether the patient had tinnitus preoperatively, 64 (56%) were reported to have tinnitus (Table 1).

**Table 1:** Patient characteristics and details of the surgical procedure

	*n* = 156
Age mean	55.2
Sex *n* (%)	
Male	55 (35.3)
Female	101 (64.7)
Op method, *n* (%)	
Stapedotomy	138 (88.5)
Stapedectomy	12 (7.7)
Operation aborted	5 (3.2)
Missing data	1 (0.6)
Surgical technique*, *n* (%)	
Drill	72 (46.2)
CO_2_	12 (7.7)
Laser CO_2_	35 (22.4)
Green laser	8 (5.1)
Laser, other	5 (3.2)
Other	49 (31)
Prosthesis, *n* (%)	
Piston Teflon/platina	120 (76.9)
Piston titan	15 (9.6)
Nitinol	3 (1.9)
Other	13 (8.3)
Missing data	5 (3.2)
Prosthesis length: mm, *n* (%)	
4	7 (4.5)
4.5	70 (44.9)
5	30 (19.2)
5.5	15 (9.6)
6	23 (14.7)
6.5	5 (3.2)
Other	1 (0.6)
Missing data	5 (3.2)
Prosthesis diameter: mm, *n* (%)	
0.4	144 (92.3)
0.6	7 (4.5)
Missing data	5 (3.2)
Anesthesia, *n* (%)	
Local	38 (24.4)
General	118 (75.6)
Tinnitus, preoperative, *n* (%)	
Yes	64 (41.0)
No	53 (34.0)
Missing data	39 (25.0)
Type of clinic, *n* (%)	
University clinic	112 (72)
County clinic	44 (28)

*Multiple options possible

### Hearing outcome

**Fig. 2:**
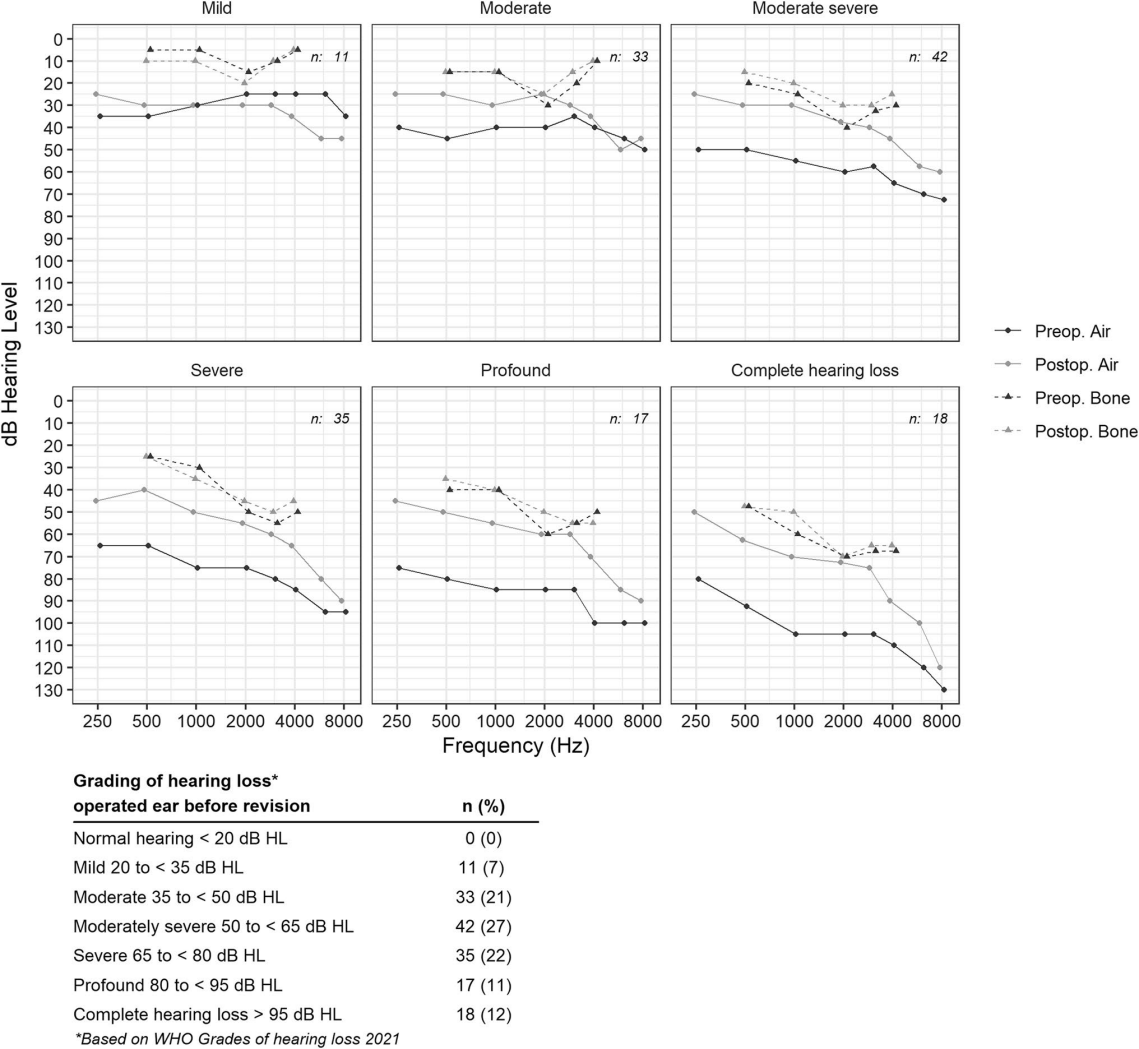
Medians of preoperative and postoperative audiometry data based on preoperative grading of hearing loss

According to the WHO grading of hearing loss, 23% of the patients had profound or complete hearing loss before surgery, and 27% had moderate to severe hearing loss (Fig. 2). The medians of pre- and postoperative audiometry data based on preoperative grading of hearing loss are shown in Fig. 2. The pure tone average for AC thresholds preoperatively was 64.4 dB HL in the study population in total. The postoperative mean hearing level was 47.3 dB HL (PTA_4_ AC). The ABG mean was 31.4 dB preoperatively and 15.6 dB postoperatively. Preoperative and postoperative hearing according to PTA_4_ AC did not differ between patients operated on at university or county clinics. A small significant difference was found in preoperative ABG between county clinics and university clinics, with larger ABG preoperatively for university clinics; however, it was not considered clinically relevant (Table 2). Forty percent had an ABG closure (≤ 10 dB), and 77% had an ABG ≤ 20 dB postoperatively. Sixty-six percent had improved hearing, defined as PTA4 AC improvement by more than 10 dB. Seventeen percent had unchanged AC hearing, and another 17% had hearing deterioration. Of these cases, six (4%) had PTA_4_ AC deterioration ≥ 20 dB (Table 2).

**Table 2:** Hearing outcome and baseline data

Mean (SD) median (IQR)	Total (*n* = 156)	University hospital (*n* = 112)	County hospital (*n* = 44)	Difference between university and county hospital *p* value*
Preoperative pure tone audiometry				
PTA_4_ AC (dB HL)	64 (22.7) 61 (31.3)	65 (23.5) 63 (31.3)	62 (20.3) 61 (32.5)	*p* > 0.30
ABG (dB)	31 (12.9) 29 (20.0)	33 (12.7) 30 (20.0)	28 (12.8) 28 (17.5)	***p* = 0.038**
Postoperative pure tone audiometry				
PTA_4_ AC (dB HL)	47 (24.2) 41(35.0)	47 (23.1) 41 (35.0)	49 (26.9) 40 (38.8)	*p* > 0.30
ABG (dB)	16 (11.6) 13 (11.3)	16 (11.5) 13 (11.3)	15 (11.9) 11 (13.8)	*p* > 0.30
Improvement PTA_4_ AC (dB)	17 (20.3) 16 (28.8)	19 (19.3) 19 (27.5)	13 (22.5) 15 (18.8)	*p* = 0.12
Change PTA_4_ BC (dB)	1 (7.3) 1 (8.8)	2 (7.0) 1 (8.8)	0 (8.0) 0 (7.5)	*p* = 0.11
Postoperative ABG *n* (%)				
≤ 10 dB	62 (39.7)	42 (37.5)	20 (45.5)	*p* > 0.30
11–20 dB	57 (36.5)	44 (39.3)	13 (29.5)	
21–30 dB	21 (13.5)	14 (12.5)	7 (15.9)	
> 30 dB	16 (10.3)	12 (10.7)	4 (9.1)	
Change in PTA_4_ AC, *n* (%)				
Improvement ≥ 30 dB	47 (30.1)	35 (31.3)	12 (27.3)	*p* = 0.30
Improvement 20–29 dB	28 (17.9)	21 (18.8)	7 (15.9)	
Improvement 10–19 dB	28 (17.9)	22 (19.6)	6 (13.6)	
Unchanged 0–9	27 (17.3)	17 (15.2)	10 (22.7)	
Deterioration 1–19 dB	20 (12.8)	13 (11.6)	7 (15.9)	
Deterioration ≥ 20 dB	6 (3.8)	4 (3.6)	2 (4.5)	

Bold denotes *p* < 0.05

*dB* decibel, *ABG* air bone gap, *PTA*_*4*_ pure tone average mean of frequencies 0.5, 1, 2 and 4 kHz, *AC* Air conduction, *BC* bone conduction, *IQR* interquartile range

*Fisher’s permutation test, Fisher’s exact test

### Complications after surgery

Eleven percent (*n* = 17) had worsened or newly developed tinnitus 1 year after surgery, as reported by the surgeon at follow-up. Five percent (*n* = 7) had persistent taste disturbance, and 3% (*n* = 4) had dizziness. No case of facial palsy was reported. When comparing university clinics and county clinics, the incidence of taste disturbance was higher for county clinics than for university clinics [12% (*n* = 5) and 2% (*n* = 2), respectively]. For tinnitus and dizziness, no differences were found (Table 3).

**Table 3:** Complications after surgery reported by the surgeon 1 year after surgery

Postoperative symptoms *n* (%)	Total	University hospital	County hospital	Difference between university and county hospital, *p* value*
Worsened or new tinnitus (*n* = 151**)	17 (11.3)	10 (9.2)	7 (16.7)	*p* > 0.30
Taste disturbance ** (*n* = 151)	7 (4.6)	2 (1.8)	5 (11.9)	***p* = 0.035**
Dizziness*** (*n* = 150)	4 (2.7)	3 (2.8)	1 (2.4)	*p* > 0.30
Facial palsy *** (*n* = 150)	0 (0.0)	0 (0.0)	0 (0.0)	–

Bold denotes *p* < 0.05

*Fisher’s exact test

**Data are missing for *n* = 5 patients

***Data are missing for *n* = 6 patients

In the database, there were two cases (1.3%) of postoperative deafness (PTA4 AC = 130 dB HL) after revision surgery. These cases had PTA AC values of 55 dB and 75 dB HL preoperatively. One case of deafness was operated on at the university clinic, and the other case was operated on at the county clinic (1:112 (0.9%) and 1:44 (2.3%), respectively, *p* value > 0.3).

### Patient-reported data

Seventy-five percent (*n* = 75) of the patients reported better to much better hearing 1 year after revision surgery. Fourteen percent (*n* = 14) reported unchanged hearing, and 6% (*n* = 6) reported worse or much worse hearing postoperatively. The patient-reported outcome regarding hearing level aligned with the audiometric change in PTA_4_ postoperatively (Table 4). When comparing county clinics with university clinics, the proportion of patients reporting better to much better hearing was larger in patients operated on in county clinics compared with university clinics [84% (*n* = 27) and 76% (*n* = 48), respectively].

**Table 4:** Patient-reported outcome 1 year after revision surgery for otosclerosis in relation to audiometry data

Hearing after surgery	Total (*n* = 95)	Change PTA_4_ AC			
	*n* (%)	Mean (SD) dB	Improvement ≥ 10 dB *n* (%)	Unchanged < 10 dB to < − 10 dB *n* (%)	Deterioration ≤ − 10 dB *n* (%)
Much better	35 (36.8)	30 (13.6)	32 (33.7)	3 (3.2)	0 (0.0)
Better	40 (42.1)	20 (15.3)	32 (33.7)	7 (7.4)	1 (1.1)
Unchanged	14 (14.7)	7 (12.4)	4 (4.2)	10 (10.5)	0 (0.0)
Worse or much worse	6 (6.3)	− 15 (31.9)	1 (1.1)	2 (2.1)	3 (3.2)

### Regression analysis

Logistic regression analyses were performed with the type of hospital (county clinic or university clinic) as a predictor for postoperative complications and postoperative hearing outcome. The type of clinic was not found to be a significant predictor of postoperative hearing outcome (ABG), tinnitus or dizziness. We found that surgery at a county clinic was a significant predictor (*p* = 0.0035) of taste disturbance postoperatively; however, the results must be cautiously interpreted given the low numeric values for the investigated endpoints, i.e., five and two cases of taste disturbance for county clinics and university clinics, respectively. Furthermore, when analyzing factors that predict a decline in hearing level after surgery, sex and preoperative ABG were found to be significant predictors, where female sex was correlated with a lower risk of postoperative decline in hearing level [*p* = 0.0045 OR 0.25 (0.09–0.65)], and a larger preoperative ABG was correlated with a lower risk of decline in hearing level [*p* = 0.0042, OR 0.94 (0.90–0.98)].

## Discussion

The hearing results following stapes revision surgery were reported to be much better in 75% of the patients, and 77% had an ABG < 20 dB after surgery in this nationwide prospective quality register-based study. No differences in hearing outcome were found between university clinics and county clinics.

Compared with previous literature on revision surgery, the hearing outcome in this study, with 77% achieving a postoperative ABG ≤ 20 dB, is comparable with several other major studies, and for ABG < 10 dB, the result in this study (44%) is slightly lower than that in other reports, where the results range between 40 and 80% [1, 2, 5, 21–28].

Regarding worsened hearing, 4% of the patients in this study had a deterioration of more than 20 dB postoperatively, and 1.3% (two cases) had postoperative deafness (1 out of 112 for university clinics and 1 out of 44 for county clinics). Compared with primary stapes surgery, these rates are significantly higher and reveal the higher risk that follows revision surgery. The rates of hearing deterioration after revision stapes surgery vary widely depending on different definitions of hearing loss. In one of the largest studies on revision surgery, including 652 cases by Vincent et al., postoperative hearing loss was reported in 2. 9% of the cases, and in another large study by de la Cruz hearing loss was reported in 7.7% of the 356 cases examined [1, 2, 5, 21–28].

Postoperative deafness is, together with persistent dizziness, the most devastating complication in otosclerosis surgery. The risk of deafness following stapes revision surgery has been suggested to be up to five times greater than in primary stapes surgery [5]. Several studies report rates of approximately 1–2% of deaf ears after stapes revision surgery [3, 5, 6]. When we compared university clinics and county clinics, we found no differences in the prevalence of hearing loss postoperatively, and one case of deafness was found in each group (1:112 (0.9%) and 1:44 (2.3%), respectively, *p* value > 0.30).

### Patient-reported data

This study includes patient-reported data on symptoms after surgery and specific questions regarding taste disturbance, dizziness, tinnitus and patients’ experience of hearing change after surgery. There are few data in the literature on these symptoms in relation to stapes revision surgery. However, for primary stapes surgery, there are studies on all tinnitus. The results from this study showed that 10% of the patients experienced newly developed or increased tinnitus after surgery. These results are similar to those of other studies after primary stapes surgery, such as the study by Ramsay et al. [29] on 246 cases of stapedectomy, which showed that 11% of the patients reported increased tinnitus postoperatively, and the study by Bagger et al. [30], which revealed that 18% of patients experienced increased tinnitus.

Regarding taste disturbance, reports on prevalence in relation to stapes revision surgery are scarce. In the literature on primary stapes surgery, the results are consistent with our findings that 4.6% of patients report persisting taste disturbance 1 year after surgery. For example, Bergling-Holm et al. reported 5% taste disturbance, and 2.9% taste disturbance was seen in the national quality database study by Strömbäck et al. [16, 31].

When comparing county clinics with university clinics, we found a significantly higher occurrence of taste disturbance for patients operated on in county clinics, 11.9 vs. 1.8%, respectively. The cause for this is uncertain, and the results should be cautiously interpreted given the low numeric values.

A factor influencing the rate of complications may be the volume of surgical procedures in the different clinics. A high case load per surgeon and the number of years of experience as an ear surgeon have been identified as important factors in the overall success rate of ear surgery, e.g., tympanoplasty [32] and stapes surgery [33]. This study, however, does not investigate the impact of the individual experience of the surgeons or case load per surgeon but rather examines hearing outcome and complication rate after stapes revision surgery on a national level comparing university clinics with higher volumes in general and county clinics with lower volumes in general.

Given the higher risk of postoperative hearing deterioration and deafness in revision stapes surgery, it is crucial to select the right cases for revision surgery. We found that a larger preoperative ABG was associated with a lower risk of postoperative hearing deterioration. In line with these findings, Sharaf et al. found that the magnitude of the preoperative ABG is an important prognostic marker for hearing outcome in the sense that a small preoperative ABG is associated with worse hearing outcome [34].

Overall, patients with large ABG and moderate to severe hearing loss preoperatively appear to have a higher chance of successful surgery; in principle, one could recommend avoiding revision surgery in patients with mild hearing loss and smaller ABG. Of course, in patients with mild hearing loss and debilitating dizziness, a revision procedure can be absolutely necessary to relieve the symptom burden.

### Study strengths and limitations

The strengths of this study are the prospectively collected data from the vast majority of surgical procedures performed in one country gathered in a quality register database rather than the more commonly reported studies from one center or one particular surgeon. Another strength is the inclusion of patient-reported data regarding both hearing outcome and postoperative symptoms; patient-reported data are very seldom found in research on stapes surgery. The limitations of the study are the lack of specific details regarding surgical indication and detailed information concerning the surgical procedure.

## Conclusions

Revision surgery for otosclerosis is a challenge for the otosurgeon, but no differences in hearing outcome between university and county clinics were demonstrated in this nationwide study. The risk of hearing deterioration and deafness is higher than in primary stapes surgery, and it revision surgery may be recommended mainly in cases with large ABG and moderate to severe preoperative hearing loss to optimize patient outcomes with respect to risks and benefits. Patient-reported outcome measures should be included in future studies on stapes surgery.

## References

[1] Vincent R, Rovers M, Zingade N (2010). Revision stapedotomy: operative findings and hearing results. A prospective study of 652 cases from the otology-neurotology database. Otol Neurotol.

[2] De La Cruz A, Fayad JN (2000). Revision stapedectomy. Otolaryngol-Head Neck Surg.

[3] Gros A, Vatovec J, Žargi M, Jenko K (2005). Success rate in revision stapes surgery for otosclerosis. Otol Neurotol.

[4] Mann WJ, Amedee RG, Fuerst G, Tabb HG (1996). Hearing loss as a complication of stapes surgery. Otolaryngol Head Neck Surg.

[5] Lundman L, Strömbäck K, Björsne A, Grendin J, Dahlin-Redfors Y (2020). Otosclerosis revision surgery in Sweden: hearing outcome, predictive factors and complications. Eur Arch Otorhinolaryngol.

[6] Babighian GG, Albu S (2009). Failures in stapedotomy for otosclerosis. Otolaryngol-Head Neck Surg.

[7] Luft HS, Bunker JP, Enthoven AC (1979). Should operations be regionalized?. N Engl J Med.

[8] Birkmeyer JD, Siewers AE, Finlayson EVA (2002). Hospital volume and surgical mortality in the United States. N Engl J Med.

[9] Flood AB, Scott WR, Ewy W (1984). Does practice make perfect? Part I: the relation between hospital volume and outcomes for selected diagnostic categories. Med Care.

[10] Redmann AJ, Yuen SN, VonAllmen D (2019). Does surgical volume and complexity affect cost and mortality in otolaryngology-head and neck surgery?. Otolaryngol-Head and Neck Surg.

[11] Mulvey CL, Pronovost PJ, Gourin CG (2015). Hospital volume and failure to rescue after head and neck cancer surgery. Otolaryngol-Head and Neck Surg.

[12] Wuthrick EJ, Zhang Q, Machtay M (2015). Institutional clinical trial accrual volume and survival of patients with head and neck cancer. J Clin Oncol.

[13] Barker II, Fred G, Carter BS, Ojemann RG, Jyung RW, Poe DS, McKenna MJ (2003). Surgical excision of acoustic neuroma: patient outcome and provider caseload. Laryngoscope.

[14] Andresen NS, Gourin CG, Stewart CM, Sun DQ (2020). Hospital volume and failure to rescue after vestibular schwannoma resection. Laryngoscope.

[15] Yung M, Oates J, Vowler S (2006). The learning curve in stapes surgery and its implication to training. Laryngoscope.

[16] Strömbäck K, Lundman L, Bjorsne A, Grendin J, Stjernquist-Desatnik A, Dahlin-Redfors Y (2017). Stapes surgery in Sweden: evaluation of a national-based register. Eur Arch Otorhinolaryngol.

[17] Available at: https://oto.registercentrum.se/in-english/the-swedish-quality-register-for-otosclerosis-surgery/p/HkkvwtQjP. Accessed 29 Mar 2022.

[18] Committee on hearing and equilibrium guidelines for the evaluation of results of treatment of conductive hearing loss. Otolaryngol–Head Neck Surg 1995; 113:186–187. 10.1016/S0194-5998(95)70103-6 7675477

[19] Watson GJ, da Cruz M (2018). Reporting in stapes surgery: are we following the guidelines?. J Laryngol Otol.

[20] Chadha S, Kamenov K, Cieza A (2021). The world report on hearing, 2021. Bull World Health Organ.

[21] Albers AE, Schönfeld U, Kandilakis K, Jovanovic S (2013). CO2 laser revision stapedotomy. Laryngoscope.

[22] Hammerschlag PE, Fishman A, Scheer AA (1998). A review of 308 cases of revision stapedectomy. Laryngoscope.

[23] Lippy WH, Battista RA, Berenholz L, Schuring AG, Burkey JM (2003). Twenty-year review of revision stapedectomy. Otol Neurotol.

[24] Luryi AL, Schettino A, Michaelides EM, Babu S, Bojrab DI, Schutt CA. Revision stapes surgery: hearing symptoms and associations with intraoperative findings and outcomes. Otolaryngol Head Neck Surg 2021:1945998211062074. 10.1177/01945998211062074 34846954

[25] Schmid P, Häusler R (2009). Revision stapedectomy: an analysis of 201 operations. Otol Neurotol.

[26] Schwam ZG, Schettino A, Babu SC, Bojrab DI, Michaelides EM, Schutt CA (2021). Outcomes in revision stapes surgery. Otolaryngol-Head and Neck Surg.

[27] Somers T, Govaerts P, de Varebeke SJ, Offeciers E (1997). Revision stapes surgery. J Laryngol Otol.

[28] Thiel G, Mills R (2011). Persistent and recurrent conductive deafness following stapedotomy. J Laryngol Otol.

[29] Ramsay H, Kdrkkdinen J, Palva T (1997). Success in surgery for otosclerosis: hearing improvement and other indicators. Am J Otolaryngol.

[30] Bagger-Sjöbäck D, Strömbäck K, Hultcrantz M (2015). High-frequency hearing, tinnitus and patient satisfaction with stapedotomy: a randomized prospective study. Sci Rep.

[31] Berling Holm K, Knutsson J, Strömbäck K (2017). Taste disturbance after stapes surgery: an evaluation of frequency, severity, duration, and quality-of-life. Acta Otolaryngol.

[32] Bedri Eh, Worku A, Redleaf M (2019). The effect of surgeon experience on tympanic membrane closure. Laryngosc Investig Otolaryngol.

[33] Brkic FF, Erovic BM, Onoprienko A (2021). Impact of surgeons' experience and the single-shot perioperative antibiotic prophylaxis on outcome in stapedotomy. PLoS ONE.

[34] Sharaf K, Grueninger I, Hilpert A (2021). Stapes and stapes revision surgery: preoperative air-bone gap is a prognostic marker. Otol Neurotol.

